# Metabolome and Essential Element Analyses of Five Underutilized European Crops Reveal Their Nutritional Properties and Potential to Diversify the European Food System

**DOI:** 10.3390/metabo15110720

**Published:** 2025-11-03

**Authors:** Mihail Angelov, Valentina Ivanova, Plamen Stoyanov, Rumen Mladenov, Tsvetelina Mladenova, Donika Gyuzeleva, Simona Zhelyazkova, Tzenka Radoukova, Krasimir Todorov, Stefka Bogdanova, Aleksandra Ivanova, Michael Wittenberg, Veselin Petrov, Tsanko Gechev

**Affiliations:** 1 Center of Plant Systems Biology and Biotechnology, 14 Knyaz Boris I Pokrastitel Str., 4023 Plovdiv, Bulgaria; 2 Department of Botany and Biological Education, Faculty of Biology, University of Plovdiv “Paisii Hilendarski”, 24 Tsar Assen Str., 4000 Plovdiv, Bulgaria; 3 Department of Bioorganic Chemistry, Faculty of Pharmacy, Medical University of Plovdiv, 15a Vasil Aprilov Str., 4000 Plovdiv, Bulgaria; 4 Department of Plant Physiology, Biochemistry and Genetics, Agricultural University Plovdiv, 12 Mendeleev Str., 4000 Plovdiv, Bulgaria; 5 Department of Molecular Biology, Faculty of Biology, University of Plovdiv “Paisii Hilendarski”, 24 Tsar Assen Str., 4000 Plovdiv, Bulgaria

**Keywords:** *Achillea millefolium*, *Agastache rugosa*, *Cercis siliquastrum*, *Crithmum maritimum*, *Mespilus germanica*, minerals, primary metabolites, secondary metabolites

## Abstract

**Background/Objectives:** People in the developed world tend to consume food that is rich in calories but lacks sufficient nutrients such as essential minerals, vitamins, and other health-promoting metabolites. At the same time, hunger and malnutrition are still problems in other countries. Therefore, various forms of micronutrient deficiencies and diseases caused by unbalanced nutrition are global issues. **Methods:** In order to elucidate the beneficial potential of alternative food sources, we employed state-of-the-art UHPLC-MS and ICP-MS technologies to perform comprehensive metabolome and metallome analyses of five edible European plants, some of which are known as underutilized crops: *Achillea millefolium*, *Agastache rugosa*, *Cercis siliquastrum*, *Crithmum maritimum*, and *Mespilus germanica*. **Results:** This study reveals valuable nutritional properties such as high levels of essential amino acids, sugars, organic acids, health-promoting secondary metabolites, and essential microelements that are important for human diet. The analyzed samples indicate that *A. millefolium*, *C. siliquastrum*, and *M. germanica* could be marked as a viable source of beneficial flavonoids. In turn, both leaves and fruits of *A. rugosa* had elevated abundances of organic acids, along with *A. millefolium* and *C. siliquastrum*. Similar results were observed for amino acids. **Conclusions:** Taken as a whole, the fruits of *C. siliquastrum* could be described as the best source for most of the identified compounds. The *M. germanica* samples were rich in mineral contents, with indications that they can supply 26% of the recommended daily intake per 100 g for K, 16% for Mg, 26% for Fe, 63% for Mn, and 89% for B. The leaves of *C. maritimum* and *A. millefolium* are also a good source of K and Mn. Interestingly, the sampled leaves of *C. maritimum* contained a very high amount of B, representing more than three times the reference nutrient value for 100 g of plant material. In conclusion, these underutilized species can be used to diversify the European food systems by enriching our diets with essential nutrients and health-promoting metabolites.

## 1. Introduction

The growing population on the planet has underlined the necessity for more productive, sustainable, and diverse cropping systems, as hunger and malnutrition remain a major challenge for many developing countries. According to the United Nations, 2.4 billion people face moderate to severe food insecurity, whereas 9.2% of the world’s population find themselves in a state of chronic hunger. A key factor in overcoming this challenge is addressing both the production and consumption aspects of agriculture and food systems. Agriculture in developing regions is often limited by over-reliance on a few staple crops, which leads to low dietary diversity and feeds malnutrition [1]. Diversification of the industry through neglected and underutilized crops can expand food sources, reduce dependency, and encourage more sustainable agricultural practices [2].

In recent years, there has been a growing interest in neglected and underutilized crops (NUCs). Despite being overlooked, these crops possess varied and rich chemical composition, offering unique flavors and high concentrations of essential micronutrients, antioxidants, and bioactive phytochemicals. As such, underutilized crops represent a significant reservoir of natural compounds with promising applications across industrial biotechnology and pharmaceutical development. Additionally, some show climate resilience and economic viability, which could make them crucial elements in strategies to combat poverty, hunger, and malnutrition [3].

Despite their potential, research into the chemical composition and phytochemical profiles of these crops remains limited. While much is known about the antioxidant properties and health benefits of common fruits, the vast majority of underutilized crops remain underexplored. This gap in knowledge prevents full utilization of these crops, hindering their incorporation into food systems and medicinal practices. On the other hand, the antioxidant activity and phytochemical content of fruits can vary significantly due to factors such as genetics, environmental conditions, and the specific plant parts used, which can also impact the safety of their consumption [4]. Meanwhile, in today’s interconnected world, where global travel and social media have made exotic foods more accessible, there is growing concern over the consumption of rare or unfamiliar plant species. Social media influencers, often without scientific expertise, explore exotic cuisines and share their experiences, with limited knowledge of the composition of the foods they promote. High-end restaurants, too, are increasingly using rare plants as decorative elements in dishes, which can come into direct contact with food and raise safety concerns. With the growing interest in wild edible plants (WEPs) and underutilized crops as food supplements for healthy diets, unraveling their complex chemical composition is crucial for the development of novel, safe, and sustainable food sources.

Untargeted comparative approaches help researchers discern conserved primary metabolism, specific metabolite patterns, and family-specific secondary metabolite profiles. They can be applied across different species [5,6], within species [7,8] or across geographical distribution in a single specie [9]. For plants with nutraceutical properties, variances in growing conditions could lead to large fluctuations in the metabolites of interest, making these types of studies of interest.

As part of this study, we explored the properties of five underutilized crops, used in traditional cuisine and folk medicine for centuries: *Achillea millefolium* (yarrow), *Agastache rugosa* (Korean mint), *Cercis siliquastrum* (Judas tree), *Crithmum maritimum* (samphire), and *Mespilus germanica* (common medlar). These edible plants have demonstrated diverse applications in nutrition, medicine, and environmental sustainability. A review of the literature indicated they could be utilized as crops because of their uses as edible plants and the lack of acute toxicity in any of the species from normal dietary use or the use of extracts.

The current work aims to build on previous studies on the chemical composition and bioactivity of these five species [10,11,12,13,14,15,16,17,18,19,20,21,22,23,24,25,26,27,28,29,30,31,32,33] using untargeted metabolomics techniques (GC/UHPLC-MS) and ICP-MS to analyze various plant materials from these underutilized species grown in Bulgaria. Our goal is to deepen the understanding of plant metabolomics and phytochemical diversity, which can aid in advancing crop breeding, uncovering novel medicinal compounds and contributing to global biodiversity preservation.

Through continued research into these underutilized crops, their full potential could be unlocked, and they could be integrated into sustainable food systems in a more informed manner.

*Achillea millefolium* (Asteraceae), commonly known as yarrow, is a resilient “weed” known for its ability to grow in a variety of environments. Yarrow has a long history of use in traditional Balkan recipes and folk medicine—the leaves in salads or other dishes, the dried flowers in teas or as seasoning—and is prized for both its flavor and healing properties [10]. *A. millefolium* contains a wealth of essential oils, characterized by a complex mix of monoterpenes and sesquiterpenes. Its chemical composition fluctuates depending on the plant’s age and morphotype. These variations enhance its wide range of therapeutic properties like anti-inflammatory and analgesic effects [11,12]. Today, yarrow’s natural antimicrobial activity is being explored as a clean-label alternative to chemical preservatives in the food industry, which is especially relevant given rising concerns over antimicrobial resistance [13]. In summary, it is a versatile plant with substantial medicinal and culinary potential; its anti-inflammatory, antimicrobial, and antioxidant properties make it a valuable asset in both natural medicine and modern food preservation. As consumer demand for natural ingredients grows, yarrow stands out as a promising sustainable solution in both health and industry.

*Agastache rugosa* (Lamiaceae) is known for its sweet anise-like flavor and fragrant leaves and flowers. It is used in cuisine, teas, and folk medicine, especially for anxiety, insomnia, and pain relief [14,15]. *Agastache* species are rich in essential oils and bioactive compounds, including flavonoids and phenolic acids, which contribute to their medicinal properties. These compounds show promise for cardiovascular health, metabolic regulation, and antimicrobial activity [16]. Notably, tilianin exhibits cardioprotective effects, while extracts also support metabolic health, with antidiabetic and anti-obesity properties [17]. This species also shows potential in cancer treatment, with cytotoxic effects on certain cancer cell lines [18,19]. Additionally, Agastache oil serves as a natural pesticide, offering a safer alternative to synthetic options [20]. These findings highlight *A. rugosa* as a valuable source of bioactive compounds with diverse medicinal and industrial applications.

*Cercis siliquastrum* (Fabaceae), commonly known as the Judas tree, is a deciduous ornamental species [21]. It is renowned for its vibrant pink flowers and heart-shaped leaves and is widely cultivated in temperate urban and garden landscapes. The plant’s young leaves, shoots, and seeds are edible and nutrient-rich, containing a variety of micronutrients, and have traditionally been consumed in salads or roasted. In traditional medicine, *C. siliquastrum* has been used to treat malaria, anemia, stress, and gastrointestinal disorders [22]. However, gaps remain in understanding its full phytochemical profile and biological activities. Recent analyses have revealed a complex array of volatile compounds and flavonoids that contribute to the tree’s antioxidant, antimicrobial, and anti-inflammatory activities [23]. Notably, myricitrin exhibits neuroprotective properties, while terpenoids like isoborneol and safranal enhance antimicrobial effects. Flavonoids such as kaempferol and quercetin also suggest anticancer potential by inducing apoptosis in cancer cells [22]. Beyond medicinal applications, *C. siliquastrum* shows promise in cosmetics and agriculture. Its UV-protective and antimicrobial compounds may be useful in natural skincare formulations and eco-friendly pesticides [24]. Furthermore, its resilience in urban environments supports biodiversity and makes it valuable for sustainable urban landscaping. Overall, *Cercis siliquastrum* is a scientifically and economically valuable species with diverse applications across health, industry, and ecological restoration.

*Crithmum maritimum* (Apiaceae), commonly known as sea fennel, is a salt-tolerant xerophyte native to coastal habitats and is gaining attention as a promising crop for biosaline agriculture due to its resilience to salinity, nutrient-poor soils, and other abiotic stresses [25]. With growing interest in sustainable agriculture, sea fennel’s potential in the agri-food sector is increasingly recognized. Historically used in Mediterranean cuisine for its refined taste and aroma, *C. maritimum* also holds value in traditional medicine and cosmetology [26]. Nutritionally, sea fennel is rich in polyunsaturated fatty acids, particularly omega-3 and omega-6, as well as essential minerals [27]. Extracts from the flowers and fruits exhibit strong antioxidant and antimicrobial activities against pathogens such as *Staphylococcus aureus* and *Candida albicans* [27]. Medicinally, *C. maritimum* has demonstrated hepatoprotective effects, including inhibiting hepatocellular carcinoma cell growth and modulating key metabolic pathways like the ones mediated by AMPK and sirtuins [28]. It shows promise in managing lipid accumulation and metabolic disorders [27]. Beyond its therapeutic potential, sea fennel has shown efficacy as a natural pesticide against *Spodoptera litura* [29] and its high chlorophyll content supports its use as a natural colorant [30]. As such, *C. maritimum* is a valuable yet underutilized plant with diverse applications in medicine, industry, and ecology.

*Mespilus germanica* (Rosaceae), known as common medlar, is an underutilized species with a rich history of use dating back nearly 30 centuries [31]. Its fruit is edible but requires post-harvest softening to reduce high tannin levels. Traditionally, all parts of the plant—bark, leaves, and fruit—have been used in folk medicine to treat ailments such as colds, digestive issues, liver disorders, and rheumatism, owing to its antiseptic, laxative, and anti-inflammatory properties [32]. Chemically, *M. germanica* is rich in essential minerals, organic acids (tartaric, citric, malic, oxalic), and sugars (sucrose, fructose, sorbitol) [33]. The fruit contains significant levels of polyphenols, flavonoids, and pectin, making it valuable for nutritional and industrial applications. For example, pectin derived from medlar is a widely used gelling agent in the food industry [34]. Recent studies highlight emerging uses, such as biodiesel production from medlar kernel oil with linoleic and oleic acids as dominant components [35]. Additionally, the plant’s waste demonstrates high efficiency in removing Ni^2+^ ions from wastewater, offering a sustainable solution for environmental remediation [36]. Still, despite its versatility, *M. germanica* remains largely overlooked. Renewed scientific interest could promote its broader application in food, medicine, energy, and environmental sectors.

## 2. Materials and Methods

### 2.1. Plant Material Collection

**Figure 1:**
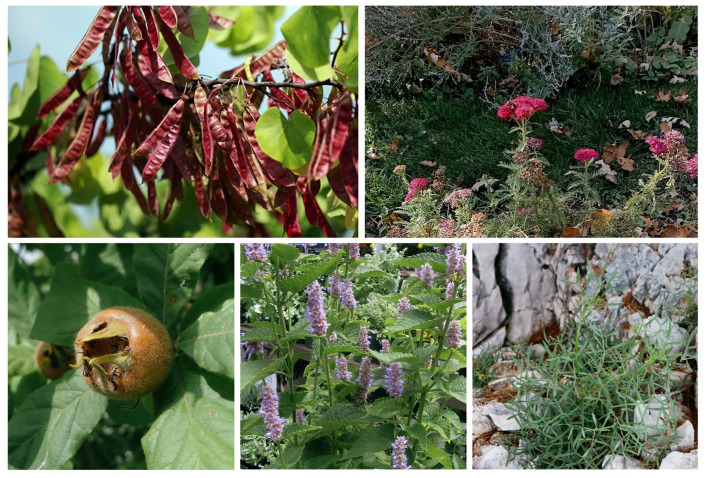
Plant species used in this study (for illustration purposes). Clockwise from top left—*Cercis siliquastrum*, *Achillea millefolium*, *Crithmum maritimum*, *Agastache rugosa*, and *Mespilus germanica*.

Material from the edible parts of the following species was collected from the following plants: *Achillea millefolium* ‘pomegranate’ (leaves and flowers), grown in the Sofia University Botanical Garden (location 42°36′ N; 23°25′ E); *Agastache rugosa* (leaves and flowers) and *Cercis siliquastrum* (fruits), grown in the Varna Botanical Garden (location 43°14′ N; 28°00′ E); and *Crithmum maritimum* (leaves), grown in the Balchik Botanical Garden (location 43°24′ N; 28°01′ E). Material from *Mespilus germanica* (fruits) was collected from plants grown in the Strandzha mountain, Bulgaria (near the Marina Reka protected area; location: 42°06′ N, 27°45′ E). Pictures of the plants and their respective organs used in this study are presented in Figure 1. Three replicates were used for each species and the different organs. All samples were flash-frozen in liquid nitrogen and ground to a coarse powder with a mortar and pestle, followed by additional homogenization to a fine powder using a bead mill (VWR, Darmstadt, Germany) at 30 Hz for 60 s. Subsequently, this material was freeze-dried at −40 °C.

### 2.2. Metabolomics Analysis

Each sample (20 mg of freeze-dried material) underwent a standard two-phase (methanol–chloroform) extraction [37]. Briefly, 750 µL of 100% MeOH (LC-MS grade; Chromasolv, Honeywell, Charlotte, NC, USA) with isovitexin internal standard was added to the plant material, and the samples were homogenized thoroughly, after which 400 µL of chloroform (HPLC grade; Chromasolv, Honeywell, Charlotte, NC, USA) was added. After homogenization on a shaker at 1200 rpm for 2 min, 800 µL of water (LC-MS grade; LiChrosolv, Merck, Darmstadt, Germany) was added to the samples and vortexed well. Finally, samples were centrifuged for 10 min at 15,000× *g*. Isovitexin, CAS: 29702-25-8 (Sigma-Aldrich, Merck, Darmstadt, Germany), was used as an internal standard, in order to compensate for technical variation [38]. The aqueous phase was then analyzed on a UHPLC-MS (ACQUITY UPLC I-Class PLUS, SYNAPT XS; Waters, Milford, MA, USA).

Chromatographic conditions: BEH C18 column (1.7 µm, 2.1 mm × 100 mm; Waters, USA), kept at 40 °C. Sampler temperature was set at 15 °C. Mobile phase A was 0.1% formic acid (LiChropur, Merck)–water (LiChrosolv, Merck), and phase B was 0.1% formic acid–acetonitrile (Chromasolv, Honeywell). Flow rate was set at 0.4 mL/min. The gradient was as follows: 0–1 min, 1% B; 1–11 min, 1–40% B; 11–13 min, 40–70% B; 13–15 min, 70–99% B; 15–16 min, 99% B; 16–17 min, 99–1% B; 17–20 min, 1% B. The injection volume was 2 µL.

Q-TOF mass spectrum conditions: an electrospray ion source (ESI) was used. Capillary voltage: 2.5 kV; sampling cone voltage: 40 V; source temperature: 120 °C; desolvation temperature: 250 °C. Cone gas flow was 50 L/h, desolvation gas flow was 600 L/h, and nebuliser gas flow was 6.5 bar. The detector was set in negative mode and resolution analyzer mode, with a continuum survey with a range of 50–1500 Da and scan time of 0.3 s. For MS/MS, a gradient fragmentation energy of 20 to 30 V was used.

Post-acquisition, data were imported into Progenesis QI (Waters/Non-Linear Dynamics, USA) and processed using default parameters. In brief, all runs were assessed for suitability as an alignment reference, performing automatic alignment. Peak picking was performed at automatic sensitivity (default setting), with no retention time limits, a fragment sensitivity of 0.2% the base peak, and all available adducts. Abundances were normalized to the sample mass and isovitexin standard. Compound data were exported and annotated using the metabolite database, available at the Center of Plant Systems Biology and Biotechnology in Plovdiv, and then referenced to publicly available fragmentation spectra databases (e.g., MassBank [39]).

Derivatization was carried out as by Lisec et al. [40] on 100 µL of dried extract using 20 mg/mL methoxyamine hydrochloride (Sigma-Aldrich, Merck, Darmstadt, Germany) in pyridine (Emsure; Merck, Darmstadt, Germany) and trimethylsilyl-N-methyl trifluoroacetamide (Restec, Pittsburgh, PA, USA). Derivatized extracts were analyzed on a TSQ9000 GCMS (Thermo Fisher Scientific, Waltham, MA, USA) following a one-microliter injection. Helium was used as carrier gas at a constant flow rate of 2 mL/sec, and gas chromatography was performed on a 30 m DB-35 column with a 0.32 mm inner diameter and 0.25 μm film thickness (Agilent Technologies, Santa Clara, CA, USA). The injection temperature was 230 °C, and the transfer line and ion source temperatures were set to 250 °C. The initial temperature of the oven (85 °C) increased at a rate of 15 °C/min up to a final temperature of 360 °C. After a solvent delay of 180 s, mass spectra were recorded at 20 scans/s with a 70–600 *m*/*z* scanning range. The chromatograms were processed in Xcalibur v.4.1.31, and peaks were annotated to an internal library of over 100 standards recently analyzed on the same instrument. In addition, prominent peaks not present in the internal library were manually annotated by matching spectra to publicly available databases (e.g., Fiehn Spectral library [41]).

Chromatograms are included in the accompanying Supplementary Data.

Only compounds annotated in all five species were included in the comparative analysis. A heatmap was generated, and PCA was performed with the help of the online software MetaboAnalyst 6.0, using the log transformation and autoscaling functions [42].

### 2.3. Metalomic Analysis

A MARS 6 microwave digestion system (CEM Corporation, Matthews, NC, USA) was used for sample preparation. The digestion was performed in PTFE vessels by adding 0.5 mL trace-metal-grade concentrated HNO_3_ (Suprapur; Merck) and 2 mL 30% H_2_O_2_ (Emsure; Merck) to 0.25 g homogenized and lyophilized samples. The samples were left for 30 min before being placed in the microwave and digested in closed vessels. Samples and method blanks were prepared and digested in a single batch and later diluted to 15 mL with reagent water (Arium Comfort I—H_2_O-I-1-TOC-T; Sartorius, Göttingen, Germany).

Measurements were performed using an inductively coupled plasma–mass spectrometer (7850 ICP-MS, Agilent Technologies, Santa Clara, CA, USA). The system was fitted with a glass nebulizer, quartz spray chamber, nickel cones, and a torch with a 2.5 mm injector, as well as an Ultra High Matrix Introduction system and ORS4 cell operating in helium (He) mode. The optimized ICP-MS operation conditions for analysis were as follows: RF power of 1600 W, plasma argon flow rate of 15.0 L/min, and nebulizer gas flow rate of 0.9 L/min. Data acquisition was performed using Mass Hunter software. ICP-MS data was imported into Microsoft Excel, and each of the detected elements was calculated using a first-order (linear) calibration curve. Detailed data for all elements is presented in a Supplementary Table.

## 3. Results

### 3.1. Metabolomics Analysis

**Figure 2:**
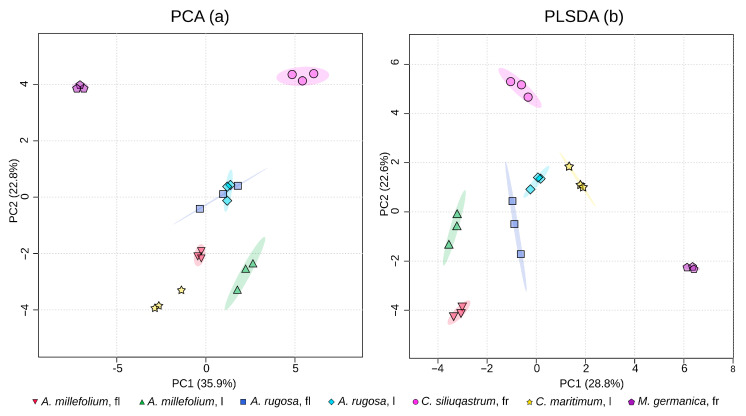
Principal component analysis plots (unsupervised, (**a**); supervised, (**b**)), indicating the clear separation of the metabolites from all five studied species and their respective organs. For the unsupervised analysis, the first and second principal components were 35.9% and 22.8%, respectively, and 28.8% and 22.6% for the supervised analysis. The plots show clear separation between all species and tissues, with the exception of *A. rugosa* leaves and flowers, which overlap in the unsupervised analysis. Tissue type is denoted as follows: l—leaves; fl—flowers; fr—fruits.

Following systematic annotation of the collected data, a comparative metabolomics analysis across the five analyzed species revealed 98 shared compounds in the data acquired via UPLC-MS and GC-MS (39 and 59, respectively). Full lists of compounds and their detection method could be seen in Supplementary Table S1. Principal component analysis (PCA) showed clear separation between all species, and in most tissues, only the *A. rugosa* inflorescences and leaves were indistinct from another (Figure 2a). However, these were separated using a supervised approach (Figure 2b).

**Figure 3:**
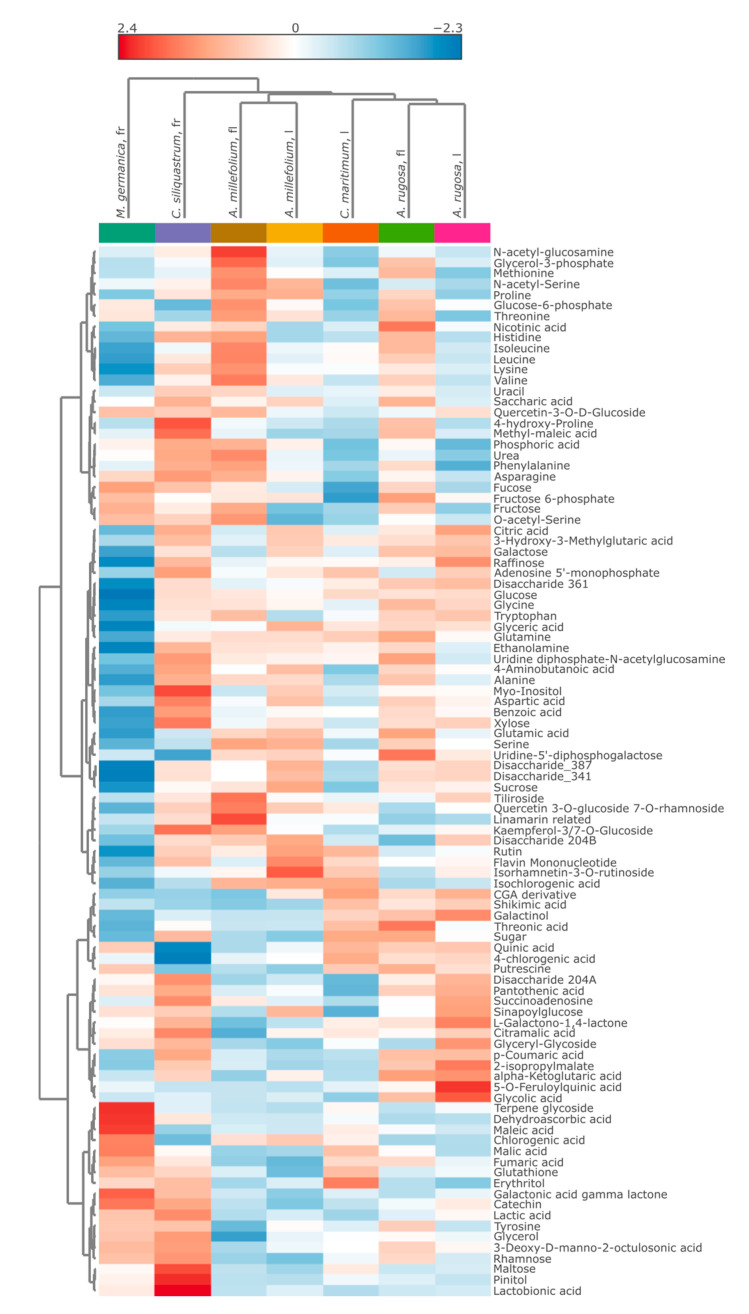
Heat map with all 98 annotated metabolites from the five edible plants.

The identified metabolites exhibited interspecies variation in their distribution patterns and relative abundances, as evident on the heatmap (Figure 3), suggesting potential ecological or physiological adaptations. Most major phytochemical classes, including flavonoids (e.g., quercetin and kaempferol derivatives), organic acids (such as citric and citramalic acids), amino acids (like asparagine, glutamine, histidine), and carbohydrate derivatives were present.

#### 3.1.1. Primary Metabolite Profiles of the Edible Tissues

For amino acids, a robust set was annotated and cross-referenced between all samples. Inflorescences of *A. millefolium* had the highest abundance in general, with a notable exception being tyrosine, which was lowest in this tissue. Opposite to it, *M. germanica* fruits had the lowest amount of most amino acids, followed by *C. maritimum* leaves. Many of these compounds have nutraceutical properties according to published data.

Asparagine was mostly present in *C. siliquastrum*, followed by *A. millefolium* inflorescences and then *M. germanica*. It can be synthesized from aspartate with asparagine synthetase, with reports of deficiencies in this enzyme leading to brain structural abnormalities and cognitive impairments [43]. Aspartate was likewise most abundant in *C. siliquastrum*, but the second and third most abundant were *A. millefolium* leaves and *A. rugosa* inflorescences, respectively. This amino acid is not an essential one, but there is an ongoing discussion on whether, as a structural homolog of glutamate, it acts as a secondary excitatory neurotransmitter in the central nervous system [43]. Glutamine is an extremely versatile amino acid that takes part in numerous biological activities—immune, metabolic, and stress response, among others [44]. The inflorescences of *A. rugosa* had the highest abundance of this compound, followed by *C. maritimum*, and both *A. millefolium* tissues. The essential amino acid histidine was present, in order of concentration, in the generative organs of *A. millefolium*, *C. siliquastrum*, and *A. rugosa*, and finally in the leaves of *C. maritimum*. Leucine, isoleucine, and tryptophan were present at the highest concentration in the inflorescences of *A. millefolium* and *A. rugosa*, while lowest in *M. germanica* fruit.

One of the annotated shared metabolites is 3-hydroxy-3-methylglutaric acid (HMG) with the highest quantities in the fruits of *C. siliquastrum*, followed by the leaves of *A. rugosa* and *A. millefolium*, and finally *A. rugosa* inflorescences and *C. maritimum* leaves.

Two other compounds, with the highest abundance in *C. siliquastrum* fruits, were lactobionic acid and myo-inositol. Both have been implicated in antioxidant and anti-inflammatory defenses [45,46,47].

Shikimic acid had the highest abundance in the leaves of *C. maritimum*. Alpha-ketoglutaric acid (AKG) was present in the highest amounts in both tissues of *A. rugosa*.

Citric acid was detected with the highest abundance in *A. rugosa* leaves, followed closely by *C. siliquastrum* fruits and *A. millefolium* leaves. Results for citramalic acid were similar, with the first and second position reversed.

Fumaric acid was highest in *M. germanica*, followed by *C. maritimum* leaves and *A. rugosa* inflorescences. *M. germanica* fruits also had high presence of dehydroascorbic acid and maleic acid.

#### 3.1.2. Secondary Metabolite Composition

Most of the detected flavonoids had high abundance in the inflorescences of *A. millefolium*, with the exception of isorhamnetin-3-O-rutinoside and rutin, most accumulated in its leaves.

One other identified compound in this group—the glycosidic flavonoid tiliroside, has a wide nutraceutical profile, due to its antioxidant and anti-inflammatory activities [48], demonstrating antidiabetic, anti-obesity, hepatoprotective, and lipid-lowering effects, partly through the modulation of adiponectin signaling and inhibition of key inflammatory pathways [49,50,51].

While most of the identified flavonoids in the analyzed fruits of *M. germanica* were among the lowest across all samples, catechin was the highest—this data supports previous work on polyphenol contents in medlar fruits [52,53].

Numerous organic acids were annotated, with a highly variable profile between species. Quinic acid and its derivatives, 4-chlorogenic acid (4-O-caffeoylquinic or cryptochlorogenic acid) and isochlorogenic acid B were the most abundant in the leaves of *C. maritimum*, followed by *A. rugosa* and *M. germanica* for the first and second and *A. millefolium* for the third. Chlorogenic acid (5-O-caffeoylquinic acid) had a different distribution, present the most in *M. germanica*, followed by in *A. millefolium* and *C. maritimum*. 5-O-feruloylquinic acid was discovered at very high levels in *A. rugosa* leaves in comparison to all other examined tissues and species. p-Coumaric acid levels were highest in *C. siliquastrum* and *A. rugosa*.

Taken as a whole, *C. siliquastrum* fruits established themselves as the richest in the sampled tissues, with the highest abundance of a majority of the annotated compounds. On the other hand, *M. germanica* showed generally lower abundances, except for catechin, which was highest in the sampled tissues. Still, while there are some trends in the distribution of the compounds among the analyzed species and tissues, there is no clear-cut “best” source of flavonoids, amino acids, saccharides, or other compounds.

### 3.2. Metalomics Analysis

The micronutrient concentration varied widely both between the studied species and between their studied botanical parts, which might depend on the habitat type (Table 1).

Our results show that *C. maritimum* has the highest Na content, with the potential to provide 3.2% of the recommended daily intake.

It is notable that both the leaves and the inflorescences of *A. rugosa* and *A. millefolium* contained significant amounts of the nutrient reference values for Mn—28–47% and 25–66%, respectively. The data shows that the latter can also be considered an excellent source of Cu, which varied between 21 and 24% in the inflorescences and leaves. A meal containing 100 g of *A. rugosa* can prove to be a good source of Co, providing 21% of the daily recommended intake.

Another interesting finding is that the sampled leaves of *C. maritimum* showed a very high amount of B, representing more than three times the reference nutrient value for 100 g of plant material.

## 4. Discussion

This study presents a comprehensive comparative metabolome and metalome analyses of five underutilized crops. The combined analysis of primary and secondary metabolites, as well as essential minerals, is a novelty of this study. The data shows unique metabolite profiles of the five species, with each one possessing specific metabolic and elemental signatures. In *A. millefolium*, we identified many metabolites, including essential amino acids and minerals that highlight the nutritional values of this species. Leaves of *A. millefolium*, in particular, are the richest in sucrose among all other species and organs, and one of the richest sources of the essential minerals K, Na, Mn, Mg, and Zn, making them a valuable source of nutrients. The leaves of *A. millefolium* are also valuable sources of beneficial flavonoids, which makes them important not only for our diet but also for our health. *A. rugosa* is known for its culinary use and nutritional values. Previous research, however, indicated the presence of valuable primary and secondary metabolites, especially compounds with potential medical applications, but lacked comprehensive metabolome and mineral profiling [14,15,16,17,18,19,20]. Here, we confirm its value and report for the first time that flowers of *A. rugosa* are the richest source of Mg, an essential element for the human diet that participates in many enzymes and biochemical processes. The areal parts of *A. rugosa* are also particularly rich in amino acids, specifically glutamine and histidine. Among the other novel findings of our study are the highest abundances of lactobionic acid and myo-inositol in *C. siliquastrum* fruits. As these compounds are implicated in antioxidant and anti-inflammatory defenses, the fruits of *C. siliquastrum* appear to be not only an important source of nutrients but also an important source of health-promoting metabolites. We also found that *C. maritimum*, known previously for its richness in polyunsaturated fatty acids and its medicinal properties [27,28,29,30,31,32,33], has the highest concentration of Na and Ca. This is to be expected, regarding its halophytic environment rich in such ions. We report here, for the first time, that *C. maritimum* is rich in B—an essential microelement that is not present in the other species. Furthermore, *C. maritimum* and *M. germanica* are the two most abundant species in glutathione: an essential antioxidant, important for maintaining the reactive oxygen species balance and for detoxification reactions in both plants and animals. We discuss some of these and other important findings below.

### 4.1. Species-Specific Levels of Primary and Secondary Metabolites

The metabolite compositional diversity is in accordance with established frameworks in plant systems, where flavonoids often mediate stress responses, while organic acids and sugars are involved in central carbon metabolism and energy homeostasis. The heterogeneity in abundance profiles suggests species-specific regulatory mechanisms, governing various biosynthetic pathways. Such variability may be the product of multiple factors—genetic, environmental, or ontogenetic—underscoring the dynamic interplay between metabolic network foundations and ecological niche adaptation.

Amino acids are fundamental in both dietary and nutraceutical aspects, being critical components for protein synthesis, metabolic regulation, and other cellular processes. Nutritionally, essential amino acids support muscle maintenance, hormone production, and immune function, with deficiencies linked to different metabolic disorders. Tryptophan is an essential amino acid that serves as a precursor to critical neurotransmitters like serotonin and melatonin, making it vital for mood regulation, sleep quality, and neurological help [54]. It is also implicated in the direct modulation of brain activity through the gut–brain axis, with leucine showing similar, but lower activity [55]. There is data suggesting that glycine could provide benefits by improving exercise capacity and supporting muscle protein synthesis and collagen production [56]. Nutraceutically, targeted supplementation can address specific health needs, enhance cognitive and athletic performance, and help with chronic disease management.

Galactinol is mostly studied through the raffinose family oligosaccharides pathway, where it acts as essential precursor and presents as a potential prebiotic, promoting the growth of beneficial bacteria and reducing the harmful bacteria present in the colon [57].

HMG, also known under the name meglutol, has the ability to lower low-density lipoprotein (LDL)—an effect observed both in vitro, and in animal and human studies [58,59,60]. It acts as a statin, inhibiting the HMG-CoA reductase (HMGCR) enzyme, and thus the synthesis of mevalonic acid—the last step of the upper mevalonate pathway, limiting the production of all downstream products, one of which is cholesterol [59]. Another intriguing point is that there are reports suggesting that long-term statin therapy leads to improvement in quality-adjusted life years in men and women aged 70 and above [61]. This might be indicative of health benefits on the dietary inclusion of compounds with statin-like activity, such as HMG.

Pinitol was found in elevated levels in the fruits of *C. siliquastrum*; given its relation to carob, its high content of this compound is not unexpected. There is substantial peer-reviewed research documenting pinitol’s nutraceutical benefits in multiple contexts—hepatoprotective, antioxidant, anti-inflammatory, and anti-osteoporotic [62,63]. Shikimic acid has various biological activities, including antioxidant, antiviral, immunomodulatory, and anti-inflammatory [64,65]. Due to its use as a precursor for the synthesis of the antiviral molecule oseltamivir (active ingredient of the medication sold under the name Tamiflu), there is research into the antiviral properties of shikimic acid itself. One study found a noted virucidal effect of shikimic acid nanoformulations against bird flu (H5N1); moreover, the presented data also points to strong immunomodulatory effects, inhibiting cytokines and nitric oxide production by approximately 50% [66].

As part of the citric acid cycle, AKG has been implicated in multiple biological activities. Its antioxidant effects are twofold. It has ROS scavenging properties, reacting directly with H_2_O_2_, thereby reducing oxidative stress by neutralizing it [67]. Along with that it can modulate the levels of important antioxidant enzymes and compounds. For instance, it can increase the activity of superoxide dismutase (SOD) and glutathione peroxidase (GPx), while lowering the levels of malondialdehyde (MDA) [67]. There is data supporting that AKG supplementation in mouse models leads to improvements in metabolic and glucose homeostasis by increasing brown adipose tissue temperature, oxygen consumption, and whole-body metabolism, significantly decreasing the activity of liver gluconeogenesis enzyme flux and ameliorating hyperglycemia [68].

Fumaric acid and its esters have a diverse profile of biological activity. A study indicates that fumaric acid has a strong inhibitory effect against bacterial and fungal cells, and proves to have selective toxicity against bacterial cells [69]. There are well-described immunomodulatory effects, which are used in the treatment of psoriasis and other autoimmune conditions; a noted potential application is for the alleviation of multiple sclerosis [70]. p-Coumaric acid is another metabolite of interest, due to its multiple documented health benefits—antioxidant, anti-inflammatory, and cardiovascular- and neuroprotective effects [71].

Flavonoids, secondary metabolites synthesized through the phenylpropanoid pathway, are a large class of polyphenolic compounds widely distributed in fruits, vegetables, and grains, recognized for their diverse phenolic structures and biological activities. Nutritionally, flavonoids are valued for their strong antioxidant properties, which help neutralize free radicals and reduce oxidative stress, thereby lowering the risk of chronic diseases such as cardiovascular disease, certain cancers, and neurodegenerative disorders [72]. Medicinally, they are known for anti-inflammatory, antiviral, and neuroprotective effects, acting through mechanisms such as the inhibition of pro-inflammatory enzymes and modulation of key cellular pathways [73]. These properties mark their potential in nutraceutical, pharmaceutical, and therapeutic contexts for disease prevention and health promotion.

Radušienė et al., 2023, noted that leaves of *A. millefolium* have a higher concentration of rutin, in comparison with the inflorescences consistently observed across multiple regions in Turkey and Lithuania [74]. Rutin is also noted for its antidiabetic activity through enhanced insulin signaling and hepatoprotective benefits by mitigating oxidative liver damage [75].

As with other flavonoids, catechins have antioxidant and anti-inflammatory properties, but they have other beneficial effects, which might prove interesting. There is growing evidence that they possess antidiabetic activity, through multiple mechanisms [76], one of which is an insulin mimetic effect. It promotes GLUT4 (glucose transporter type 4) transport into cells, enhancing the uptake of glucose and glycogen synthesis via glycogen synthase, thus helping to reduce the effects of insulin resistance [76,77]. Another interesting property of catechin is its anti-ulcer activity, for which there are two reported mechanisms. Its antioxidant properties help alleviate damage to gastric mucosa [78], and along with that, it has antibacterial activity, reducing the cell count of *Helicobacter pylori* in gerbils [79]. While the elimination of *H. pylori* is not total, it is similar to antibiotic monotherapy; thus, it might prove to be an interesting co-therapeutic agent. It should be noted that some catechins, especially epigallocatechin gallate (EGCG), could pose a health risk if their consumption is excessive, so the current recommendation of the European Food and Safety Administration (EFSA) is to limit intake to 800 mg per day [80].

Based on these data, *A. millefolium, C. siliquastrum*, and *M. germanica* could be marked as a viable source of beneficial flavonoids, with the choice of species depending on the compound of interest. While, in general, *C. maritimum* is low in the discussed flavonoids, it has the potential of being used as a source of isorhamnetin-3-O-rutinoside and rutin, utilizing areas with higher salinity, which might be unsuitable for other cultures. Given that *A. rugosa* inflorescences and leaves present low contents of all flavonoids, their utility as a source of that group of compounds seems limited.

Quinic acid and its derivatives, chlorogenic acid, 4-chlorogenic acid, and isochlorogenic acid B, have antioxidant and anti-inflammatory activity, through the attenuation of the secretion of inflammatory mediators such as TNF-α, IL-1β, and IL-6 [81,82].

It is known that consumer choices can be influenced by the changing needs regarding health and environmental and ethical considerations. The evaluation of the metalomic profile of these five species could help us obtain a more comprehensive understanding of those underutilized crops as a potential food source.

### 4.2. Elemental Analysis Reveals Species Rich in Essential Nutrients

Although Na is an essential nutrient and an important factor in neurotransmission and cell function, salt contributes to the increased risk of cardiovascular diseases [83]. However, the analyzed samples were well below the maximum permissible threshold of 2000 mg.

According to Regulation No 1169/2011, values higher than 15% of the nutrient reference values supplied by 100 g of product are regarded as significant [84]. From a public health perspective, several international institutions have issued a list of nutrient reference values expressed per 100 g of fresh plant material [85,86].

In considering that the daily reference intake of K is 2000 mg, *C. maritimum* and *A. millefolium* leaves, as well as the fruits of *M. germanica*, can provide 16 to 25.9% of the recommended daily intake per 100 g, thus marking them significant. The medlar fruits could supply 16% for Mg, 26% for Fe, 63% for Mn, and 88% for B.

### 4.3. General Limitations of Metabolome and Elemental Analyses

Metabolome and elemental analyses together create a comprehensive picture of nutritional values and health benefits of plant crops. However, we shall bear in mind that plant metabolomes of a single species can vary depending on geographical location, growth conditions, and environmental factors (type of soil, temperature, humidity, biotic stressors, etc.) [87,88,89,90]. This is also related to the respective changes in transcriptomes, which induce the subsequent metabolome reconfigurations. Such variations, observed for both primary and secondary metabolites, are fundamental limitations when interpreting metabolome data. In our study, we used near-optimal growth conditions provided in the Bulgarian botanical gardens. In other locations, under more fluctuating environmental conditions, certain metabolites may deviate from the values we are reporting here. Nevertheless, our cross-species comparison is a useful basis for the identification of potential crops with high levels of particular metabolites and minerals.

## 5. Conclusions

Now, more than ever, there is a growing demand for healthy diet enriched with all essential nutrients and elements. Therefore, diversifying food sources and embracing the potential of underutilized crops’ environmental and health benefits could be a step closer to achieving zero hunger, enhancing both food security and ecological well-being. With the appropriate focus on crop improvement and sustainable utilization, these promising species might play a role in promoting food sovereignty, health and sustainability in the future. This study focused on five such edible plants, previously shown to be rich in nutrients, minerals, vitamins, and other health-promoting substances, and characterized in greater detail by their chemical composition. Comparatively, *Agastache rugosa* and *Mespilus germanica* are well known in Korean and European cuisine, respectively, and are used in various dishes or as fresh fruits, the other three species are underutilized and can be used to supplement our diet. We recommend growing these species as crops and using them more intensively to diversify our food systems.

## Figures and Tables

**Table 1:** Mineral content (mg/kg DW) and calculated values of nutrient reference values per 100 g fresh plant material (%NRV). For values below the limit of detection (LOD), the %NRV cannot be calculated and is thus marked as N/A, with the exception of aluminum, for which EU regulation 1169/2011 does not specify as NRV.

Species and Tissue	K			Na		
	mg/kg	RSD	%NRV	mg/kg	RSD	%NRV
*Crithmum maritimum*, l	2661.37	8.4	16	527.85	3.17	3.17
*Cercis siliquastrum*, fruit	1402.14	0.8	8.4	3.34	1.6	0.02
*Agastache rugosa* Kuntze, fl	1858.11	0.8	11.1	9.34	0.4	0.06
*Agastache rugosa* Kuntze, l	893.62	0.9	5.4	1.18	4.7	0.01
*Achillea millefolium* ‘pomegranate’, fl	2407.66	0.3	14.4	54.89	0.7	0.33
*Achillea millefolium* ‘pomegranate’, l	3734.50	0.7	22.4	350.58	1	2.1
*Mespilus germanica*, fr	4319.00	4.1	25.9	45.30	0.27	0.27
	K LOD = 0.0450			Na LOD = 0.0285		
**Species and Tissue**	**Mg**			**Zn**		
	mg/kg	RSD	%NRV	mg/kg	RSD	%NRV
*Crithmum maritimum*, l	222.20	7.1	7.1	0.92	0.5	1.1
*Cercis siliquastrum*, fruit	157.45	0.8	5	0.96	2.8	1.2
*Agastache rugosa* Kuntze, fl	333.49	0.5	10.7	3.95	2.7	4.7
*Agastache rugosa* Kuntze, l	217.96	0.2	7	3.46	1.5	4.1
*Achillea millefolium* ‘pomegranate’, fl	153.22	0.9	4.9	4.59	0.8	5.5
*Achillea millefolium* ‘pomegranate’, l	294.91	1.0	9.4	3.92	2.2	4.7
*Mespilus germanica*, fr	506.90	0.4	16.2	3.26	6.6	3.9
	Mg LOD = 0.0161			Zn LOD = 0.0347		
**Species and Tissue**	**Ca**			**Fe**		
	mg/kg	RSD	%NRV	mg/kg	RSD	%NRV
*Crithmum maritimum*, l	493.48	7.4	7.4	4.78	2.1	4.1
*Cercis siliquastrum*, fruit	65.55	0.7	1	1.96	1.6	1.7
*Agastache rugosa* Kuntze, fl	209.64	0.9	3.1	3.28	0.7	2.8
*Agastache rugosa* Kuntze, l	333.38	0.7	5	3.74	1.7	3.2
*Achillea millefolium* ‘pomegranate’, fl	49.54	2	0.7	7.66	1.2	6.6
*Achillea millefolium* ‘pomegranate’, l	153.33	1.6	2.3	13.34	0.5	11.4
*Mespilus germanica*, fr	156.50	4.1	2.3	30.6	1.1	26.2
*Crithmum maritimum*, l	Ca LOD = 0.0248			Fe LOD = 0.0401		
**Species and Tissue**	**Mn**			**B**		
	mg/kg	RSD	%NRV	mg/kg	RSD	%NRV
*Crithmum maritimum*, l	7.89	0.4	47.4	4.47	1.3	357.6
*Cercis siliquastrum*, fruit	1.16	2.5	7	below LD	2.2	N/A
*Agastache rugosa* Kuntze, fl	4.67	2.9	28	below LD	5.6	N/A
*Agastache rugosa* Kuntze, l	7.79	0.9	46.7	below LD	0.8	N/A
*Achillea millefolium* ‘pomegranate’, fl	4.18	2	25.1	below LD	3.1	N/A
*Achillea millefolium* ‘pomegranate’, l	10.97	0.6	65.8	below LD	3.8	N/A
*Mespilus germanica*, fr	156.50	2.3	63	1.11	2.98	88.8
	Mn LOD = 0.0564			B LOD = 0.1156		
**Species and Tissue**	**Cu**			**Al**		
	mg/kg	RSD	%NRV	mg/kg	RSD	%NRV
*Crithmum maritimum*, l	0.28	0.4	3.4	3.88	0.8	N/A
*Cercis siliquastrum*, fruit	0.55	0.8	6.6	1.13	7.9	N/A
*Agastache rugosa* Kuntze, fl	0.87	0.4	10.4	2.37	1.3	N/A
*Agastache rugosa* Kuntze, l	0.51	2.3	6.1	3.58	8.9	N/A
*Achillea millefolium* ‘pomegranate’, fl	1.75	1.6	21	4.14	4	N/A
*Achillea millefolium* ‘pomegranate’, l	2.03	1.9	24.3	7.16	6.3	N/A
*Mespilus germanica*, fr	0.3	1.2	3.6	below LD	5.2	N/A
	Cu LOD = 0.0509			Al LOD = 0.0521		
**Species and Tissue**	**Co**			**Cr**		
	mg/kg	RSD	%NRV	mg/kg	RSD	%NRV
*Crithmum maritimum*, l	0.003	2.7	0.8	below LOD	1.8	N/A
*Cercis siliquastrum*, fruit	below LOD	25.5	N/A	below LOD	4.8	N/A
*Agastache rugosa* Kuntze, fl	0.089	3.2	21.4	below LOD	7.2	N/A
*Agastache rugosa* Kuntze, l	0.022	9.6	5.3	below LOD	3.1	N/A
*Achillea millefolium* ‘pomegranate’, fl	0.028	10.8	6.6	below LOD	9.4	N/A
*Achillea millefolium* ‘pomegranate’, l	0.012	9.3	2.9	below LOD	5.4	N/A
*Mespilus germanica*, fr	below LOD	4.3	N/A	below LOD	14.32	N/A
	Co LOD = 0.0014			Cr LOD = 0.0237		

## Data Availability

The UHPLC-MS chromatograms, as well as all other data, are available upon request.

## Supplementary Materials

The following supporting information can be downloaded at https://www.mdpi.com/article/10.3390/metabo15110720/s1, Table S1: Full lists of compounds and their detection method.

## Abbreviations

The following abbreviations are used in this manuscript:

AKG	Alpha-ketoglutaric acid;
GPx	Glutathione Peroxidase;
HMG	3-hydroxy-3-methylglutaric acid;
MDA	Malondialdehyde.
